# Effect of Octacalcium Phosphate on Osteogenic Differentiation of Induced Pluripotent Stem Cells in a 3D Hybrid Spheroid Culture

**DOI:** 10.3390/biomimetics10040205

**Published:** 2025-03-26

**Authors:** Yuki Sugai, Ryo Hamai, Yukari Shiwaku, Takahisa Anada, Kaori Tsuchiya, Tai Kimura, Manami Tadano, Kensuke Yamauchi, Tetsu Takahashi, Hiroshi Egusa, Osamu Suzuki

**Affiliations:** 1 Division of Craniofacial Function Engineering (Division of Biomaterials Science and Engineering), Tohoku University Graduate School of Dentistry, Sendai 980-8575, Japan; 2 Division of Oral and Maxillofacial Reconstructive Surgery, Tohoku University Graduate School of Dentistry, Sendai 980-8575, Japan; 3 Institute for Materials Chemistry and Engineering, Kyushu University, Fukuoka 816-8580, Japan; 4 Division of Pediatric Dentistry, Tohoku University Graduate School of Dentistry, Sendai 980-8575, Japan; 5 Division of Molecular and Regenerative Prosthodontics, Tohoku University Graduate School of Dentistry, Sendai 980-8575, Japan

**Keywords:** hybrid spheroids, iPS cell, octacalcium phosphate, inorganic ions, osteogenic differentiation

## Abstract

Octacalcium phosphate (OCP) has been shown to exhibit an osteogenic property and, therefore, has been utilized recently as a bone substitute, clinically. However, the stimulatory capacity for induced pluripotent stem (iPS) cells is not known. This study investigated whether OCP enhances osteoblastic differentiation of three-dimensionally cultured spheroids of iPS cells compared to hydroxyapatite (HA) and β-tricalcium phosphate (β-TCP). Mouse iPS cells were mixed with smaller (less than 53 μm) or larger (300–500 μm) sizes of calcium phosphate (CaP) granules and cultured in a laboratory-developed oxygen-permeable culture chip under minimizing hypoxia for up to 21 days. Osteoblastic differentiation was estimated by the cellular alkaline phosphatase (ALP) activities. The degree of supersaturation (DS) with respect to CaP phases was determined from the media chemical compositions. Incubated CaP materials were characterized by Fourier transform infrared spectroscopy (FTIR) and X-ray diffraction (XRD). The culture promoted well the formation of hybrid spheroids of CaP materials and iPS cells regardless of the type of materials and their granule sizes. The ALP activity of OCP was about 1.5 times higher than that of β-TCP and HA in smaller granule sizes. FTIR, XRD, and DS analyses showed that larger OCP granules tended to hydrolyze to HA slightly faster than smaller granules with time while HA and β-TCP materials tended to remain unchanged. In conclusion, the results suggest that OCP enhances the osteogenic differentiation of iPS cells more than HA and β-TCP through a mechanism of hydrolyzing to HA. This inherent material property of OCP is essential for enhancing the osteoblastic differentiation of iPS cells.

## 1. Introduction

Octacalcium phosphate (OCP), an inorganic material showing a bone regenerative capacity, has begun to be used as a bone substitute material for human maxilla and mandible bone defects with dental implant placement [1,2]. The reason behind this is that OCP shows a higher osteoconductivity compared to hydroxyapatite (HA) materials [3,4]. OCP tends to biodegrade and form new bone more than a typical biodegradable material, β-tricalcium phosphate (β-TCP) [5,6,7]. OCP is known to be a precursor for HA formation from aqueous solution [8,9] and has been considered a precursor of bone apatite crystals [10]. Chemical studies suggested that OCP exists as a cluster that theoretically leads to HA formation from amorphous calcium phosphate (ACP) [11,12,13,14], which is known to be another precursor that matures to HA [15], and that OCP has been shown to appear in chemical reactions [16]. The presence of OCP was identified in human and rat bone tissues by high-resolution transmission electron microscopic (HRTEM) observation [17].

From the observation at the site where OCP initiates new bone formation through its implantation, it was clarified that osteoblasts form a new bone matrix directly onto the surface of OCP implanted under microscopic and electron microscopic levels [3,7,18]. It was also apparent that osteoclast-like multinucleated giant cells directly resorb OCP implanted under the microscopic level [7,18]. Since it was predicted that OCP directly stimulates cellular activities based on these in vivo observations, we developed several in vitro analytical methods and clarified the following findings: (1) OCP enhances early osteoblast differentiation of mouse bone marrow-derived ST−2 stromal cells [4] in a dose-dependent manner in comparison with HA [18]. (2) Osteoclast formation is increased from the coculture of mouse bone marrow-derived osteoblasts with bone marrow macrophages without the addition of the osteoclast differentiation factor receptor activator of NF-kappaB ligand (RANKL), through increasing the expression of RANKL in osteoblasts compared to HA [18]. (3) OCP enhances differentiation of mesenchymal stem cells (MSCs) (IDG-SW3 cell line) toward osteocytes more than HA and β-TCP [18]. OCP and HA accumulated leptin receptor (Lepr)-expressing bone marrow-derived MSCs, which are known to exist near blood vessels in the bone marrow and are committed to differentiate into osteocalcin (OC)-positive osteoblastic cells, but OCP enhanced osteoblast differentiation around the material compared to HA [7]. (4) OCP promotes osteoblast differentiation during the formation of a hybrid spheroid consisting of MSCs (D1 cell line) and the material particles under an oxygen supply more than HA and β-TCP [6]. The enhancement of these cellular activations could primarily be accelerated by the unique chemical properties of OCP that induce concentration gradients of Ca^2+^ and inorganic phosphate (Pi) ions [18]. The stimulatory or regulatory capacity of OCP has also been found in bone tissue-related cells, such as chondrogenic cells [18], tendon stem/progenitor cells [19], and human umbilical vein endothelial cells (HUVECs) [18]. However, although OCP was shown to display a stimulatory capacity for such mineralized tissue-related cells, including the precursor cells in osteoblasts [18] and osteocytes [7,18] from MSCs, it is not fully clear what stage of the osteoblast differentiation lineage is activated by OCP.

OCP consists of a structure composed of an apatite layer stacked alternately with a hydrated layer; therefore, it has a structure closely related to HA [9,20,21]. Since OCP is a metastable phase in a physiological environment, it tends to hydrolyze to HA and undergo a phase conversion in vivo [3,4]. In fact, the X-ray diffraction analyses of OCP retrieved from the implanted sites (both bony site [4] and subcutaneous site [22]) confirmed the progressive structural changes of OCP into an apatitic structure. Because the conversion of OCP to HA is thermodynamically favored, it is likely to advance spontaneously and irreversibly once initiated [23,24]. During the phase conversion from OCP to HA, Ca^2+^ uptake and inorganic phosphate (Pi) ion release occur continuously [20,25]. Ca^2+^ promotes cell adhesion [26,27] and proliferation [28]. Pi ions affect osteoblast mineralization and apoptosis [29,30]. The concentration gradients of Ca^2+^ and Pi ions, due to the OCP hydrolysis, and the concentration range of these inorganic ions induced by OCP, are associated with osteoblastic differentiation and osteocyte differentiation [7,18]. The stimulatory capacity of OCP observed in vitro was reproduced in in vivo bone formation by OCP, which suggested that the phase conversion from OCP to HA plays an essential role in bone formation through cell differentiation [4].

An induced pluripotent stem (iPS) cell is a type of pluripotent stem cell, which can be generated from a somatic cell [31]. iPS cells display self-renewal and pluripotency in three germ layers [32]. Studies have been conducted to utilize iPS cells for bone regeneration, including studies on the types of scaffold materials [33,34,35,36] and the effect of the surface microstructure [37], mechanical stress [38], or oxygen concentration [39,40]. iPS cells have also been utilized for screening and elucidating biological functions [41,42].

We investigated in animal experiments a correlation between bone formation capacity and the MSC differentiation ability of calcium phosphate (CaP) materials (OCP, HA, and β-TCP) by forming MSC spheroids including CaP materials (OCP, HA, β-TCP) in a three-dimensional (3D) environment that partially mimics the living body environment under a controlled oxygen concentration [6]. In the spheroid formation by 3D culture, it is known that the cell necrosis in the center of the spheroid occurs due to insufficient oxygen supply [43,44]. To overcome this issue, we developed an oxygen-permeable polydimethylsiloxane (PDMS) culture device (Oxy chip) [45]. The Oxy chip made it possible to yield uniform spheroids with a narrow size distribution and prevent hypoxia in the core of the spheroids and subsequent central necrosis, proved by human hepatoma HepG2 cells using a hypoxia marker [45]. According to this culture device, the spheroid formation of hepatocytes [45] and MSCs, and the above-mentioned hybrid spheroid formation of MSCs with a CaP material, can be relatively easily formed without the occurrence of necrosis [6]. From these study results, we hypothesized CaP materials, including the OCP material, may have a role in the differentiation of iPS cells into osteoblast-like cells when iPS cells are cultured in a similar 3D environment in vitro.

In this study, we investigated the osteogenic differentiation potential of OCP in comparison with HA and β-TCP using osteoblast-like cells derived from iPS cells. iPS cells established from mouse gingival fibroblasts were used as the cell source because the gingival-derived iPS cells have been shown to exhibit a higher mineral deposition capacity than that of mouse MSCs during osteogenic differentiation [46], which may lead to an analysis of the osteogenic differentiation potential in the osteoblastic differentiation lineage, simulating the in vivo initiation of bone formation.

## 2. Materials and Methods

### 2.1. Preparation of Oxy Chip and Non-Oxy Chip for 3D Cell Culture

Spheroid culture chips were fabricated as previously described [45]. Briefly, a polydimethylsiloxane negative mold (25 × 25 × 8 mm) was treated by using a PIB−10 Ion Bombarder (Vacuum Device, Ibaraki, Japan) and then incubated in 4% Pluronic F−127 (Sigma–Aldrich, St. Louis, MO, USA) solution for 24 h. The base and curing agent (Silpot 184, Dow Corning Toray, Co. Ltd., Tokyo, Japan) were poured into the negative mold and cured at 70 °C for 1 h to obtain the PDMS replica having multiple cavities (512 wells, 1.00 mm in diameter, 1.05 mm pitch, 1.06 mm in depth, 25 × 25 mm section). The PDMS replica, with and without a custom-made acrylic resin tray, was used for the Oxy chip and non-Oxy chip, respectively.

### 2.2. iPS Cell Culture

**Figure 1:**
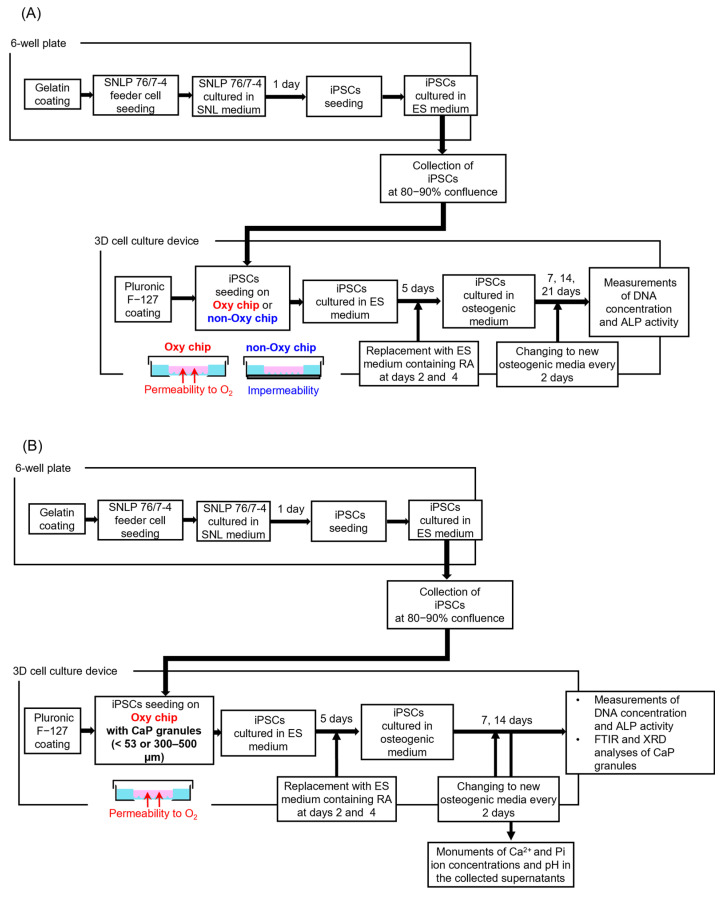
Flowchart of 3D cell culture experiments. (**A**) Three-dimensional iPSC culture with and without an oxygen supply using the 3D cell culture devices of the Oxy chip and non-Oxy chip, respectively. (**B**) Culture of hybrid spheroids of CaP granules (<53 μm or 300–500 μm) and iPSCs on the Oxy chip.

Figure 1A shows a flowchart of 3D cell culture of iPSC with and without an oxygen supply. Mouse gingiva-derived iPS cells (iPSCs) [46] were used in the present study. The culture of iPSCs was performed according to previous reports [31,46]. Briefly, the mouse gingiva-derived iPSCs were maintained in a 6-well plate in ES medium consisting of Dulbecco’s modified Eagle’s medium (DMEM; 4.5 g/L glucose w/o sodium pyruvate, Fujifilm Wako Pure Chemical Co., Osaka, Japan) supplemented with fetal bovine serum (FBS; Biosera, Cholet, France), L-glutamine (Fujifilm Wako Pure Chemical Co.), 1% penicillin/streptomycin (PS; Nacalai Tesque Inc., Kyoto, Japan), non-essential amino acids (Invitrogen, Waltham, MA, USA), and mercaptoethanol (Invitrogen) in 5% CO_2_ and a 95% air atmosphere under humidified conditions at 37 °C. Inactivated SNLP 76/7–4 feeder cells were seeded on the gelatin-coated 6-well plate at 3.0 × 10^5^ cells/mL. The inactivated SNLP 76/7–4 feeder cells were incubated in SNL medium consisting of DMEM (4.5 g/L glucose w/o sodium pyruvate, Fujifilm Wako Pure Chemical Co.) supplemented with 10% FBS (Biosera), L-glutamine (Fujifilm Wako Pure Chemical Co.), 1% PS (Nacalai Tesque Inc.) in 5% CO_2_ and a 95% air atmosphere under humidified conditions at 37 °C for 1 day. The iPSCs were seeded on the feeder cell-cultured 6-well plate with a gelatin coating. The iPSCs were incubated in the ES medium in 5% CO_2_ and a 95% air atmosphere under humidified conditions at 37 °C. The cells were collected at 80−90% confluence after the cultivation in the ES medium.

### 2.3. Three-Dimensional Culture of iPSC on Oxy Chip and Non-Oxy Chip

The culture chips were sterilized by autoclaving and then dried by heating at 200 °C for 3 h. After the assembling of the sterilized chips, 2 mL of 4% Pluronic F−127 solution was added to prevent cell adhesion to the surface of the chips, and they were incubated for 6 h. The chips were washed twice with ES medium. The proliferated iPSCs were seeded onto the Oxy and non-Oxy chips at a cell number of 1.0 × 10^6^ cells per culture chip. On days 2 and 4, the medium was replaced with ES medium supplemented with 1 μmol/L retinoic acid (RA: Fujifilm Wako Pure Chemicals Co.). From day 5, the ES medium was replaced with osteogenic medium consisting of α- modified minimum essential medium (α-MEM; Nacalai Tesque Inc.), 10% FBS (Biosera), 100 nM water-soluble dexamethasone (Sigma-Aldrich, St. Louis, MO, USA), 10 mM β-glycerophosphate disodium salt hydrate (Sigma-Aldrich), 50 μM L-ascorbic acid 2-phosphate (Sigma-Aldrich), and 1% PS (Fujifilm Wako Pure Chemicals Co.). The osteogenic medium was changed every 2 days. The incubation periods were set to 7, 14, and 21 days to investigate the expression of osteogenic indication at the early stage that eventually leads to osteogenesis. The rationale of the analysis period is based on studying the spheroid diameters after immersion in the osteogenic medium (2, 7, 11, and 17 days) and the osteogenic differentiation after immersion in the osteogenic medium (7, 14, and 21 days), respectively.

### 2.4. Culture of iPSCs in the Presence of CaP on Oxy Chip

#### 2.4.1. Preparation of CaP Granules

OCP was prepared through the wet synthesis method by mixing calcium acetate solution and sodium hydrogen phosphate solution under supersaturation with respect to OCP and HA at pH 5−6, by maintaining the degree of supersaturation with respect to HA and OCP in the range of approximately 1 × 10^18^ and 4 × 10^11^, respectively, which was calculated at 25 °C [3]. The precipitate of OCP in the solution was isolated by filtration. The isolated OCP was washed with ultra-pure water several times and dried at 105 °C for 24 h. Sintered HA and β-TCP were purchased from APACERAM: PENTAX (Tokyo, Japan) and OSferion: OLIMPUS TERUMO BIOMATERIALS (Tokyo, Japan), respectively. These CaP were ground and passed through standard testing sieves using under 200 mesh and 16 to 32 mesh sizes to prepare granules with diameters of less than 53 μm and 300–500 μm, respectively. The granules were sterilized at 120 °C for 2 h. The rationale for using these granule diameter sizes was based on the previous studies that indicated that the granule size with a 300 to 500 μm diameter was the most widely studied size to demonstrate the osteogenic potential of OCP, while the osteoconductivity was influenced to some extent by the granule size when it was compared to the sizes ranging from 53 to 300 μm, 300 to 500 μm, and 500 to 1000 μm in murine bone defects [1,3,4,7,18]. The osteogenic capacity of CaP materials with 300 to 500 μm diameters was compared with those materials having a granule size of less than 53 μm, which was the size established previously by the studies performing cellular experiments and serum protein adsorption tests [18,47].

#### 2.4.2. Culture of iPSC Spheroids with CaP Granules with Different Diameters

Figure 1B shows a flowchart of the culture of hybrid spheroids of CaP granules (<53 μm or 300–500 μm) and iPS on the Oxy chip. iPSCs were maintained in the ES medium in 6-well plates in 5% CO_2_ and a 95% air atmosphere under humidified conditions at 37 °C. iPSCs in the amount of 1.0 × 10^6^ cells, collected from the ES medium, were mixed with OCP (1 mg), HA (5 mg), and β-TCP (2 mg) granules with a diameter of 300–500 μm or less than 53 μm in 3 mL of ES medium. The mixtures were seeded on the Oxy chips coated with Pluronic F−127 and incubated in 5% CO_2_ and a 95% air atmosphere under humidified conditions at 37 °C. On days 2 and 4 of incubations, culture media were replaced with ES media containing 1 μmol/L of retinoic acid. On day 5, ES media were replaced with the osteogenic media. iPSCs with CaP granules were cultured on the Oxy chips for 7, 14, and 21 days in the osteogenic media in 5% CO_2_ and a 95% air atmosphere under humidified conditions at 37 °C. The osteogenic media were changed every 2 days.

### 2.5. Morphological Observation of iPSC Spheroids in 3D Culture

The morphologies of iPSC spheroids cultured on the Oxy chip and non-Oxy chip were observed by using a photomicroscope (Leica DFC300 FX, Leica Microsystems Japan, Tokyo, Japan). The average diameter of spheroids (n = 3) was measured according to the procedure reported by a previous study [45].

### 2.6. Measurements of DNA Concentrations and Alkaline Phosphatase (ALP) Activities of Spheroids

Spheroids were collected at 7, 14, and 21 days after the induction in osteogenic media. The spheroids were collected by washing with PBS using a plastic pipette from the culture chips. The collected spheroids were suspended in a 0.2% Triton X−100 solution and homogenized using a multi-bead shocker (Yasui Machine Co., Ltd., Osaka, Japan) (1700 rpm, 10 s.) under liquid nitrogen conditions. The DNA content was determined using a Quant-it PicoGreen dsDNA Kit (Invitrogen) and a microplate reader (DTX800, Beckman Coulter, Pasadena, CA, USA). DNA concentrations were used as an indicator of the living cells, analyzed previously through the estimation of the lactate production and oxygen consumption rate (OCR) cultured comparably on the Oxy chip and non-Oxy chip. The ALP activity was measured by a LabAssay ALP kit (Fujifilm Wako Pure Chemical Co.) according to the manufacturer’s instructions. The ALP activity was normalized using DNA amounts determined using the Pico Green kit. ALP activity was used as an indicator of the initiation toward osteogenic differentiation of MSCs based on the previous studies that indicated that the ALP activity of MSC spheroids had a correlation with bone formation by implanting CaP materials in mouse calvaria defects [6].

### 2.7. Measurement of Ion Concentrations in Osteogenic Medium

The culture media were collected and changed to new media every 2 days in the cultivations of iPSCs with CaP granules on the Oxy chips. In order to analyze the OCP hydrolysis to HA in the culture environment, the OCP granules with diameters of less than 53 μm and 300–500 μm were also immersed in the osteogenic media at 1 mg/3 mL in the absence of iPSCs. The media with OCP granules were incubated in 5% CO_2_ and a 95% air atmosphere under humidified conditions at 37 °C for 14 days. The medium was changed every 2 days. The concentrations of Ca^2+^ and Pi ions in the collected incubated osteogenic media were determined using the commercially available kits of Calcium E and Phosphor C tests (Fujifilm Wako Pure Chemical Co.), respectively. The pH of the media was also measured using a pH electrode (9616S−10D, HORIBA, Ltd., Kyoto, Japan) at 37 °C.

### 2.8. Calculation of Degree of Supersaturation (DS) with Respect to CaP in Osteogenic Medium

The degree of supersaturation (DS) with respect to OCP, HA, and DCPD in the culture media was estimated using Equation (1):

(1) $$\mathrm{DS}={\left(\frac{\mathrm{IP}}{K_{\mathrm{sp}}}\right)}^{\frac{1}{\nu }}$$

where IP, K_sp_, and ν are ionic activity products, the solubility product constant with respect to each CaP at 37 °C, and the number of ions in CaP, respectively. The K_sp_ values used for HA, OCP, and DCPD were 7.36 × 10^−60^ (mol/L)^9^ [48], 2.51 × 10^−49^ (mol/L)^8^ [49], and 2.77 × 10^−7^ (mol/L)^2^ [50], respectively. DS values = 1.0, <1.0, and >1.0 indicate saturation, undersaturation, and supersaturation, respectively. The analytical results for Ca^2+^ and Pi concentrations and pH were used for the calculation of DS values. In the calculation, three mass balance values for Ca^2+^, Mg^2+^, and Pi ions [51,52] were considered at ion strength I = 150 mM. In addition, the presence of HCO_3_^−^ and ion pairs of CaH_2_PO_4_^+^, CaHPO_4_^0^, MgHPO_4_^0^, CaHCO_3_^+^, and MgHCO_3_^+^ were assumed in the supernatants for the calculation of DS with respect to CaP phases [51,52].

### 2.9. Structural Analysis of CaP Incubated in the Spheroids

The cell lysates containing the granules of CaP in Triton X−100 were washed with ultra-pure water and centrifuged three times. The washed granules separated from the cell lysates were frozen at −20 °C and lyophilized for 48 h. Fourier transform infrared (FT-IR) spectra of granules diluted in KBr were measured by FT-IR spectroscopy (FTIR; FT/IR−6300, JASCO Co., Tokyo, Japan) over the range of 1800–400 cm^−1^ with a resolution of 4 cm^−1^.

The crystal phase of the granules collected from the spheroid cultures was analyzed by X-ray diffraction (XRD; MiniFlex 600; Rigaku Electrical Co., Ltd., Tokyo, Japan). The XRD patterns of granules before and after the cultivations were measured using monochromatic CuKα radiation (40 kV and 15 mA) at a rate of 3.0°/min and step 0.02° intervals from 2θ = 3 to 60°. The International Centre for Diffraction Data (ICDD) was used to identify the crystal phases of OCP (00–079−0423), HA (00–009−0432), and β-TCP (00–009−0169).

### 2.10. Statistical Analysis

All cell culture experiments were performed as three independent experiments and analyzed statistically. Results are expressed as mean ± standard deviation (SD). A value of *p* < 0.05 was considered statistically significant. Tukey–Kramer multiple comparison analyses were carried out using software (Statcel 4, OMS Publishing Inc., Saitama, Japan).

## 3. Results

### 3.1. Morphological Observations of iPSCs on Oxy Chip and Non-Oxy Chip

**Figure 2:**
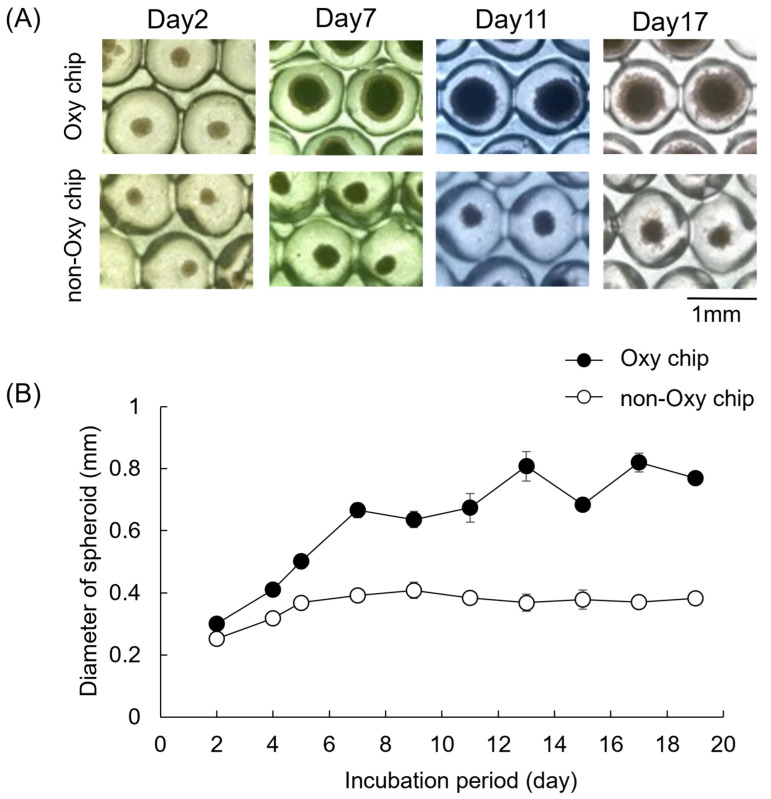
Optical microscope images and diameter of iPSC spheroids cultured on the Oxy chip and non-Oxy chip (**A**). The cell aggregate can be seen near the center of the culture chip (**B**). Bars in the images represent 1 mm.

Figure 2A shows light-microscopic images of spheroid formation on the culture chips. A single spheroid was formed in each cavity of both Oxy chips and non-Oxy chips. The cells cultured on both chips formed spheroids within 2 days (Figure 2A). There were no differences in the required time for the spheroid formation between the Oxy chip and the non-Oxy chip. In the Oxy chip, the spheroids tend to be larger up to day 7. After day 7, the spheroid size did not increase significantly. On the other hand, the diameter of the spheroids was smaller in the non-Oxy chip group than in the Oxy chip group after 7 days of cultivation. Figure 2B shows the average diameter of spheroids cultured on the Oxy chip and non-Oxy chip. The diameter of the spheroid gradually increased with the incubation period of up to 7 days on the Oxy chip. There was little change in the size of the spheroids on the non-Oxy chip after day 5. The diameters of the Oxy chip and non-Oxy chip were 684 and 378 μm, respectively, at day 15.

### 3.2. DNA Concentration and ALP Activity of iPSCs Cultured on Oxy Chip and Non-Oxy Chip

**Figure 3:**
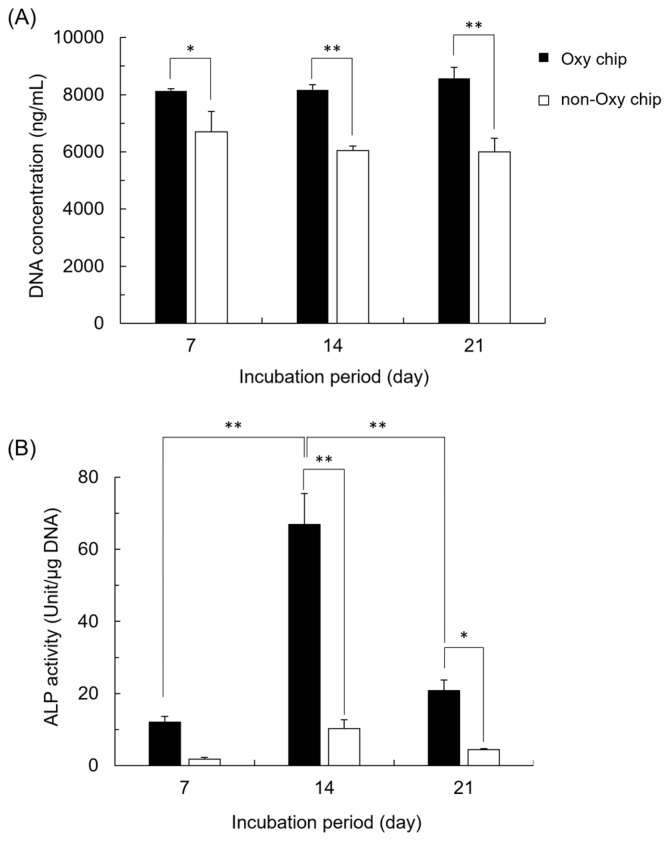
DNA concentrations (**A**) and ALP activities (**B**) of iPSCs cultured on the Oxy chip and non-Oxy chip in the osteogenic media on days 7, 14, and 21 (* *p* < 0.05, ** *p* < 0.01).

Cell proliferation on both chips was measured by the DNA concentration (Figure 3A.). Although there was little change in the cell number during the 7–21 days on each chip, when the two chips were compared, there were significant differences at all time points (days 7, 14, and 21). As shown in Figure 3B, the ALP activity, an osteoblastic differentiation marker at the early stage, of the cells cultured on the Oxy chip was significantly higher in the Oxy chip group than in the non-Oxy chip group at 7, 14, and 21 days. On day 14, the ALP activity of cells cultured on the Oxy chip was about 6.5 times higher than that of cells cultured on the non-Oxy chip.

### 3.3. Morphological Observations of iPSCs Cultured with Various CaP Granules on Oxy Chip

**Figure 4:**
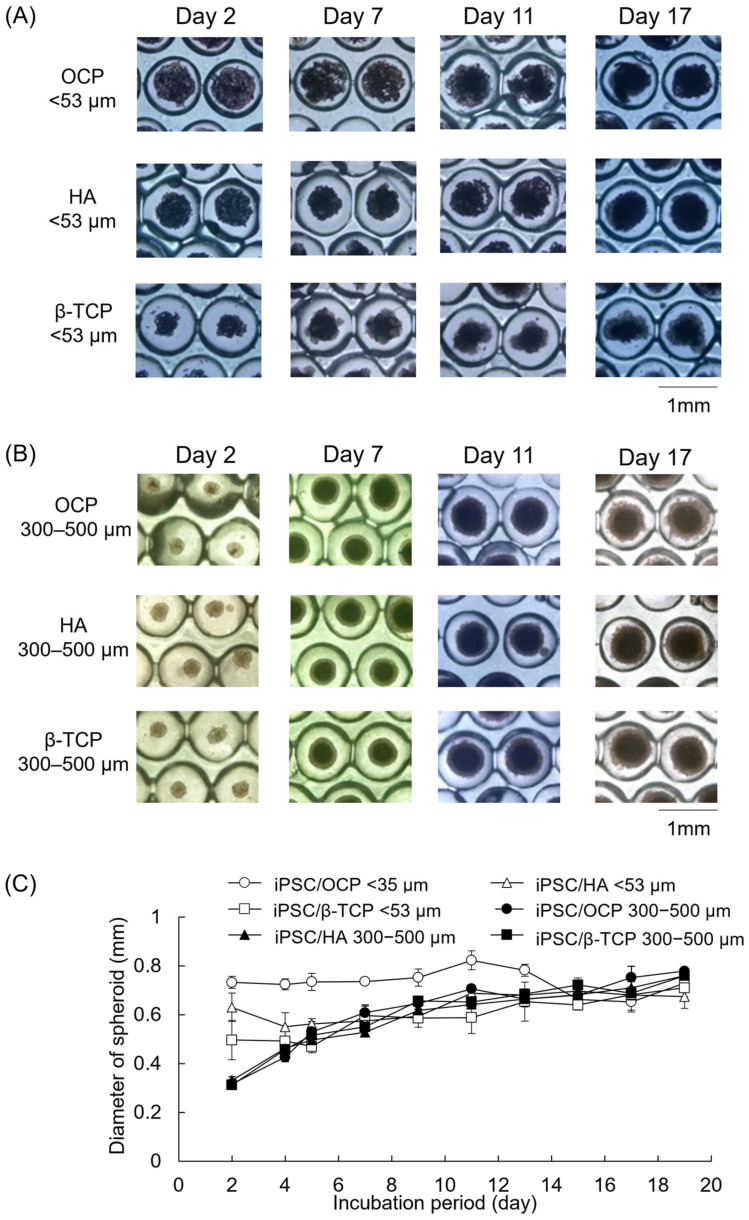
Optical microscope images of iPSCs cultured on the Oxy chip in the presence of OCP, HA, and β-TCP granules with diameters of <53 μm (**A**) and 300–500 μm (**B**). Diameters of iPSC spheroids cultured in the presence of CaP granules with diameters of <53 μm and 300–500 μm (**C**). The inclusion of CaP granules affects the contrast difference compared to those of the iPSC culture in Figure 2.

The morphologies of iPSCs were observed during the incubations on the Oxy chip in the presence of CaP granules with diameters of less than 53 μm and 300–500 μm (Figure 4A, B). In the culture with the smaller granules, the iPSCs seemed to be aggregated around the CaP granules, although these aggregates were dispersed at day 2. Part of the aggregates connected at day 7, and then, iPSCs formed larger spheroids including the smaller CaP granules. In contrast, iPSCs cultured in the presence of the CaP granules with a diameter of 300–500 μm formed spheroids via cell–cell contact. However, the number of granules included in the spheroids seemed to be lower in the 300–500 μm groups than in the <53 μm groups regardless of the crystal phase of CaP. The size of spheroids was measured using microscope images (Figure 4C). The size of spheroids increased with incubation periods until day 7 and then maintained regardless of the granular size. Although the size of spheroids seems to tend to be smaller in the 300–500 μm groups than in the <53 μm groups at the earlier stage of incubations, the size of spheroids became similar between <53 μm groups and 300–500 μm groups at the later stage of incubation.

### 3.4. DNA Concentration and ALP Activity of iPSCs Cultured with Various CaP Granules

**Figure 5:**
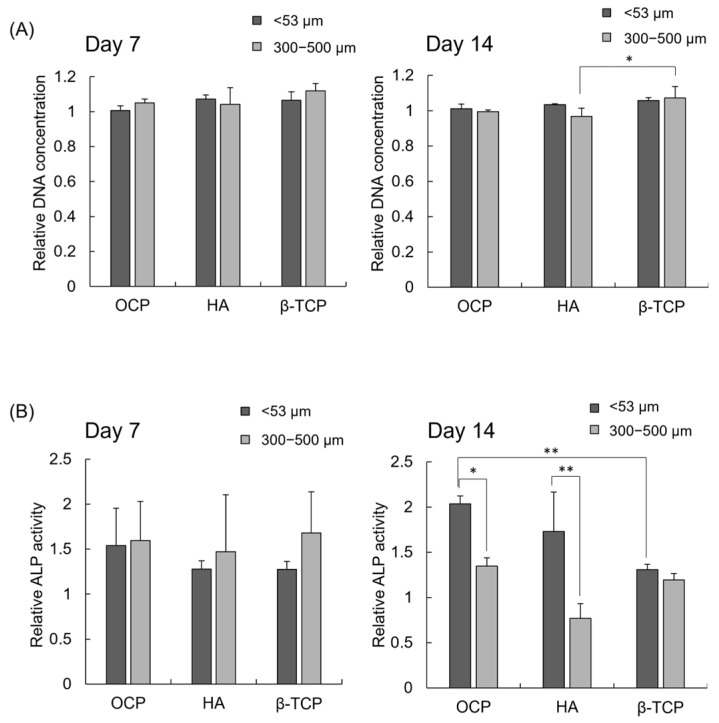
Relative DNA concentrations (**A**) and ALP activates (**B**) for iPSCs cultured in the presence of CaP granules with diameters of <53 μm and 300–500 μm on the Oxy chip normalized by control at days 7 and 14 (* *p* < 0.05, ** *p* < 0.01).

The cell proliferation of iPSCs cultured in the presence of CaP granules with different diameters was determined by the DNA concentration normalized by the control group (Figure 5A). At day 7, the relative DNA concentrations in the HA < 53 μm and β-TCP < 53 μm groups were slightly higher than in the OCP < 53 μm group, although there was no significant difference. The relative DNA concentration in the β-TCP 300–500 μm group tended to increase compared to the OCP 300–500 μm and HA 300–500 μm groups at days 7 and 14. A significant difference was observed between the HA 300–500 μm and β-TCP 300–500 μm groups at day 14. Furthermore, the DNA concentration slightly increased in the 300–500 μm group compared to the <53 μm groups after the incubation with OCP and β-TCP at day 7, and it had a similar value for the 300–500 μm and <53 μm groups at day 14. The DNA concentration tended to be lower in the HA 300–500 μm group than in the HA < 53 μm group at day 14.

The osteoblastic differentiation of the iPSC spheroids was estimated by the ALP activities normalized by the control (Figure 5B). The ALP activity in the OCP < 53 μm group tended to be higher than that in the HA < 53 μm and β-TCP < 53 μm groups at day 7. The ALP activities had similar values among the CaP phases in the presence of 300–500 μm granules at day 7. There was no significant difference on day 7. At day 14, ALP activities significantly increased in the <53 μm groups compared to the 300–500 μm groups after the incubations with OCP or HA. However, there was no significant difference between the < 53 μm group and 300–500 μm group for β-TCP at day 14. In addition, the ALP activity in the OCP < 53 μm group was the highest in all groups for <53 μm granules, and in the HA < 53 μm group, it was higher than that in the β-TCP < 53 μm group at day 14. A significant difference was observed between the OCP < 53 μm and β-TCP < 53 μm groups at day 14. The ALP activities in the OCP and β-TCP 300–500 μm groups tended to increase compared to the HA 300–500 μm group at day 14, although there was no significant difference.

### 3.5. Changes in Ion Concentrations and DS with Respect to Various CaP Materials in Culture Media

**Figure 6:**
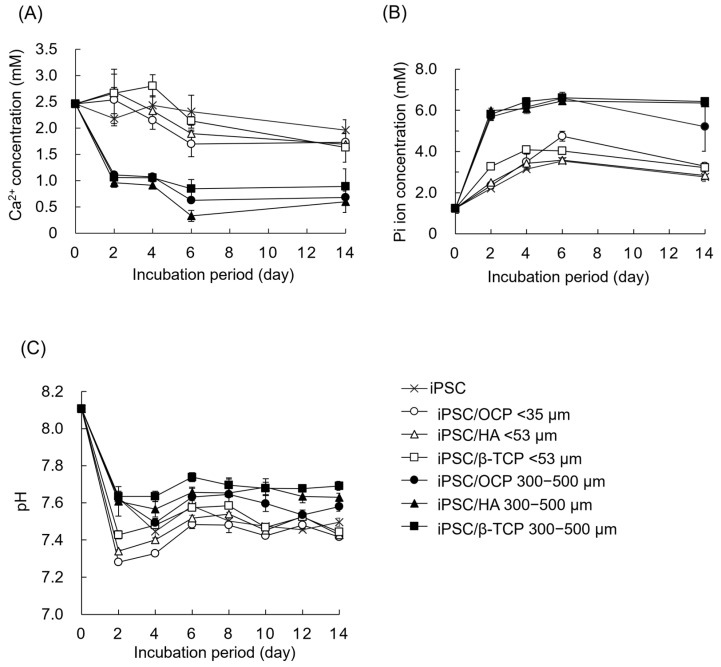
Changes in Ca^2+^ (**A**) and Pi concentrations (**B**) and pH (**C**) in the osteogenic media after the incubations of iPSCs in the presence of CaP granules with diameters of <53 μm and 300–500 μm on the Oxy chip.

The Ca^2+^ and Pi concentrations and pH in the culture media were determined after the incubations of iPSCs in the presence of CaP granules with a diameter of <53 μm or 300–500 μm (Figure 6). The Ca^2+^ concentration was higher in the <53 μm groups than in the 300–500 μm groups after the incubations of iPSCs in the presence of each CaP material. The Ca^2+^ concentration slightly increased after the incubations for 2 or 4 days and then decreased in the media incubated with CaP granules with a diameter of <53 μm. The Ca^2+^ concentration in the media with granules with a diameter of <53 μm decreased in the following order until day 6: OCP < HA < β-TCP. After the incubation of granules with a diameter of 300–500 μm, the Ca^2+^ concentration decreased from day 0 to day 6 and then maintained. At each incubation period for granules with a 300–500 μm diameter, the Ca^2+^ concentration decreased in the following order: HA < OCP < β-TCP.

The Pi concentration increased from day 0 to day 4 or 6 and then tended to maintain in the culture media regardless of CaP phases and granular size. The Pi concentration was higher in the 300–500 μm groups than in the <53 μm groups after the incubation of each CaP material. The values of Pi ion concentrations were similar between the CaP granules with a diameter of 300–500 μm. In the media incubated with the <53 μm size of granules, the Pi concentration was higher for β-TCP than for OCP and HA from day 0 to day 4. However, the Pi concentration was higher for OCP than for HA and β-TCP on day 6.

The pH values in all groups decreased from day 0 to day 2 and then tended to maintain. The pH values were lower in the <53 μm groups than in the 300–500 μm groups regardless of CaP phases. At each incubation period for granules with 300–500 μm or <53 μm of diameter, pH tended to decrease in the following order: OCP < HA < β-TCP.

The DS with respect to various CaP materials in the media was calculated by the measurement of Ca^2+^ and Pi ion concentration and pH (Table 1). The DS values indicated that the original medium was supersaturated with respect to HA and OCP, and saturated with respect to DCPD. Although the DS value with respect to HA was higher compared to OCP in the media before and after the incubations, the DS values with respect to HA and OCP decreased with incubation periods regardless of the CaP phases and granular size. The values of the DS with respect to HA tended to increase in the <53 μm groups compared to the 300–500 μm groups in the media incubated with each CaP phase. After the incubations of granules with a diameter of <53 μm, the values of the DS with respect to HA and OCP for the β-TCP group were the highest in the CaP phases, and they were higher in the HA group than in the OCP group at each incubation period. In the case of the incubations in the presence of granules with a diameter of 300–500 μm, the values of the DS with respect to HA and OCP were higher in the β-TCP group than in the OCP and HA groups at each incubation period. In addition, the DS values with respect to HA and OCP were increased in the OCP < 53 μm group compared to the HA < 53 μm group at each incubation period.

The ion compositions in the supernatants of culture media were also measured after the incubations of OCP granules with diameters of <53 μm and 300–500 μm without iPSCs (Table 2). The Ca^2+^ and Pi ion concentrations decreased after the incubations of OCP regardless of granule size. The Ca^2+^ concentration tended to be higher in the OCP < 53 μm group than in the OCP 300–500 μm group at each incubation period. Pi ion concentration was also slightly higher in the OCP < 53 μm group than in the 300–500 μm group on day 2. The DS with respect to HA and OCP decreased after the incubations of OCP granules. The DS values indicate that the medium was more supersaturated with respect to HA compared to OCP. Although the DS with respect to HA was of a similar order for the OCP < 53 μm and 300–500 μm groups at day 2, it decreased in the OCP 300–500 μm group compared to the OCP < 53 μm group at day 14.

### 3.6. FTIR Analysis of CaP Granules Incubated with iPSC Spheroids

**Figure 7:**
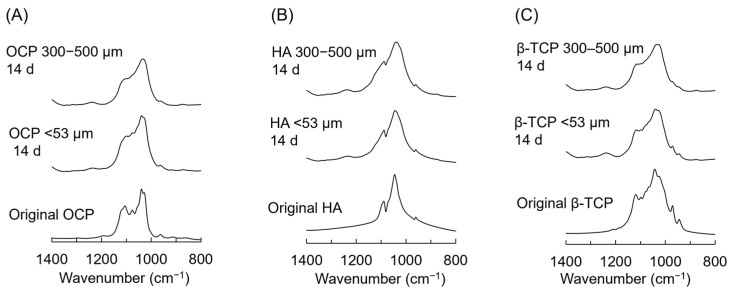
FTIR spectra of OCP (**A**), HA (**B**), and β-TCP granules (**C**) with diameters of < 53 μm and 300–500 μm after the incubations with iPSCs on the Oxy chip at day 14.

The chemical structure of various CaP granules before (original) and after the incubations were analyzed by FTIR (Figure 7). The absorption bands corresponding to ν_3_ PO_4_ and ν_3_ PO_4_/HPO_4_ were observed in the spectrum of the original OCP at 1035 and 1040 cm^−1^, respectively. The bands attributed to ν_3_ HPO_4_(5) and ν_3_ HPO_4_(6) were also detected in the spectrum of OCP at 1101 and 1123 cm^−1^, respectively [53]. In the spectrum of the original HA, the sharp bands of ν_3_ PO_4_ at 1046 and 1090 cm^−1^ appeared [54]. The bands of peaks attributed to ν_3_ PO_4_ at 1038, 1087, and 1120 cm^−1^ were detected in the spectrum of the original β-TCP [55]. After the incubations with iPSCs at day 14, the intensities of these bands were obscured in the spectra of OCP, HA, and β-TCP with a diameter of 300–500 μm compared to OCP, HA, and β-TCP with a diameter of <53 μm, respectively.

### 3.7. XRD Patterns of CaP Granules Incubated with iPSC Spheroids

**Figure 8:**
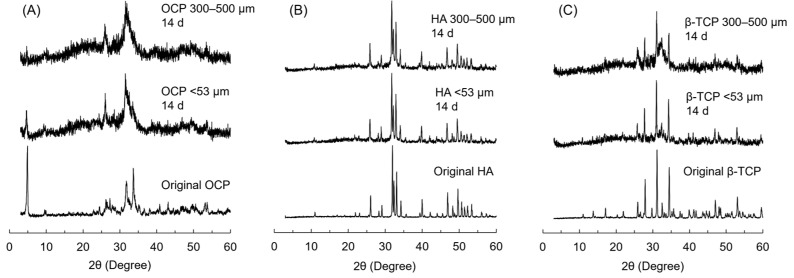
XRD patterns of OCP (**A**), HA (**B**), and β-TCP granules (**C**) with diameters of <53 μm and 300–500 μm after the incubations with iPSCs on the Oxy chip at day 14.

The crystal phase of CaP granules with different granular sizes before (original) and after the incubations at 14 days were analyzed by XRD (Figure 8). In the XRD patterns of OCP, the typical diffractions corresponding to (1 0 0), (0 1 0), (0 0 2), ($\bar{5}$ 3 0), and (7 0 0) of OCP were detected at 2θ = 4.7, 9.8, 26.2, 31.7 and 33.6°, respectively (Figure 8A). After the incubations, the intensities of (1 0 0) and (7 0 0) decreased, and diffractions attributed to (0 0 2) and (3 0 0) of HA were newly detected at 2θ = 26.0 and 32.9°, respectively, regardless of the granular size. However, the intensities of (1 0 0) and (7 0 0) were lower in the OCP 300–500 μm diameter group compared to the OCP < 53 μm group. The diffractions attributed to (1 0 0), (0 0 2), (2 2 1), and (3 0 0) of HA were detected at 2θ = 10.8, 26.0, 31.8, and 32.9°, respectively, and no peaks corresponding to other CaP phases were detected in the patterns of HA after the incubations for both granular sizes (Figure 8B). The diffractions corresponding to (2 1 4), (0 2 10), and (2 2 0) of β-TCP were detected at 2θ = 27.8, 31.0, and 34.4°, respectively, in the original β-TCP granules (Figure 8C). After the incubations, the new diffractions corresponding to HA appeared at 26.0, 31.8, and 33.0°, while the diffractions of β-TCP remained in both granular sizes. The intensities of new HA diffractions were higher in the β-TCP 300–500 μm group than in the β-TCP < 53 μm group.

## 4. Discussion

The previous studies showed that oxygen tension affects the survival and differentiation of MSCs in 2D culture [56,57,58]. Oxygen tension has been considered to have a much greater influence on 3D cell aggregates compared to the monolayer cell culture and, in fact, has been shown to contribute to the MSC differentiation in the spheroid form [59] and in the hybrid form with CaP materials [6]. However, the effect of the materials on iPSC-derived MSCs has not been elucidated. The results of the present study suggest that supplying oxygen and providing the homogeneous incorporation of OCP granules inside the iPSC aggregation may promote osteoblastic differentiation in the 3D cell culture, as shown by analyzing the ALP activity of the spheroids. iPSCs formed spheroids in the 3D cell cultures, and the proliferation of iPSCs increased on the Oxy chip compared to the non-Oxy chip (Figure 2 and Figure 3A). Although the ALP activity of the iPSC spheroids showed a peak in both 3D cultures at day 14, it was significantly higher on the Oxy chip than on the non-Oxy chip (Figure 3B). In the 3D cell culture using the Oxy chip in the presence of granular CaP materials (OCP, HA, and β-TCP) with a diameter of <53 μm or 300–500 μm, the smaller granules seemed to be incorporated homogeneously in the spheroids (Figure 4). The OCP < 53 μm material significantly increased the ALP activity of spheroids, rather than the HA and β-TCP < 53 μm material, as well as the OCP 300–500 μm material (Figure 5B). The tendency is consistent with the previous results, which were estimated by the similar spheroid 3D culture of these CaP granules and MSCs [6]. However, there were no differences in ALP activity among CaP materials with a diameter of 300–500 μm. The FTIR, XRD, and chemical analyses of culture media (Figure 7 and Figure 8, Table 1) indicated that changes in ion concentration via the hydrolysis of OCP < 53 μm could stimulate iPSCs in the spheroid to promote the osteoblastic differentiation at an early stage.

In this study, the diameter of spheroids in the Oxy chip group was larger than that in the non-Oxy chip group, and the amount of DNA was significantly increased in the Oxy chip group compared to the non-Oxy chip group, although there was almost no change in DNA content after day 7 in the Oxy chip culture. This suggests that the continuous supply of oxygen to iPS cell spheroids may enhance cell proliferation in the 3D culture condition with the Oxy chip.

A significant increase in ALP activity was observed in the Oxy chip group compared to the non-Oxy chip group, indicating that osteoblast differentiation was promoted. This suggests that an oxygen supply may promote osteoblast differentiation of iPS cell spheroids, similar to the effect of culturing mouse bone marrow-derived MSC lines in a previous study [6]. ALP is used as an undifferentiated marker for ES and iPS cells [60,61,62] as well as an osteoblast differentiation marker [63,64,65,66,67,68,69,70]. In the present study, ALP activity was higher on days 14 and 21 than on day 7 after the induction of differentiation, suggesting that the increase in ALP activity was due to the promotion of osteoblast differentiation of iPS cells rather than undifferentiated cells. This can be further confirmed by comparing the expression of osteoblast differentiation markers in the middle to late stages.

Manokawinchoke et al. reported that hypoxia promoted osteoblast differentiation of iPSCs [39], whereas our results showed contradictory results to this report. This discrepancy may be attributed to the differences between 2D and 3D cultures. The previous report also showed that MSC spheroids with a relatively large size differentiated into a chondrogenic lineage due to hypoxic conditions present in the core of the spheroids in an osteogenic differentiation medium [71]. In our previous study, we demonstrated that when hepatocytes were cultured in a 3D environment on non-Oxy chips, the partial pressure of oxygen in the medium decreased rapidly, leading to a significant increase in the percentage of necrotic areas within spheroids due to oxygen deprivation at the center. In the present study, the density of cells in the center of spheroids formed through 3D culture was much higher compared to that in 2D culture, thereby creating a hypoxic environment. However, osteoblastic differentiated iPS cells were unable to survive under hypoxic conditions, leading to suppressed cell proliferation and osteoblast differentiation. This suggests that the Oxy chip group was more advantageous for the osteoblastic differentiation of iPS cells compared to the non-Oxy chip group.

Based on the enhancement of osteoblastic differentiation of iPSC spheroids on the Oxy chip, the effects of CaP materials with different granular sizes on the osteoblastic differentiation of iPSC spheroids were examined using the Oxy chips. OCP granules with a diameter of <53 μm increased the ALP activity of iPSCs compared to HA and β-TCP granules with a diameter of <53 μm at day 14 (Figure 5). CaP particles forming the aggregation with stem cells, such as MSCs and ESCs, promote osteoblastic differentiation of these cells in 3D cell cultures [6,59,72,73]. In the present study, the formation of iPSC spheroids, including the CaP granules, was observed (Figure 4). Previous literature reported that the incorporation of OCP granules with a diameter of <53 μm increases the osteocalcin gene expression of MSC spheroids more than that of HA and β-TCP granules with a diameter of <53 μm in vitro [6]. The solubilities of CaP materials increase at 37 °C and pH 7.4 in the following order: HA < β-TCP < OCP [20,74,75]. The lower Ca^2+^ and higher Pi ion concentrations induced by OCP provide a suitable environment for the osteogenesis of bone marrow-derived MSCs and stromal cells on the 2D and 3D cell cultures via the hydrolysis reaction of OCP, depending on its solubility [6,7,18]. Our previous study reported that a lower Ca^2+^ concentration induces osteoblastic differentiation of stromal cells via the p38 signaling pathway [18]. The higher Pi concentration also enhances the osteoblastic differentiation of MSCs via the extracellular Pi transporter Pit 1 [76]. The chemical analysis showed that OCP < 53 μm material decreased Ca^2+^ and increased Pi compared to HA and β-TCP < 53 μm material in the 3D culture of iPSCs (Figure 6). The hydrolysis of OCP < 53 μm material in the 3D culture of iPSCs was confirmed by FTIR and XRD analyses (Figure 7 and Figure 8). Thus, the progress of hydrolysis of OCP incorporated in the iPSC spheroids could be involved in the enhancement of osteoblastic differentiation of the cells.

The FTIR spectra (Figure 7), XRD patterns (Figure 8), and decreasing DS with respect to HA and OCP (Table 1) indicated that the hydrolysis of OCP progressed in the 300–500 μm groups compared to the <53 μm groups. The obscurity of the FTIR spectra was also observed in the HA and β-TCP 300–500 μm groups compared to the HA and β-TCP < 53 μm groups, respectively, after the incubations of iPSCs (Figure 7). These analytical results suggested that the low level of crystalline apatite [22,77,78,79] could be formed partially on the HA and β-TCP in the larger granule sizes on the 3D culture of iPSCs. However, there is a general consensus that the solubilities of CaP increase with decreasing particle size (increasing specific surface area). Previous studies reported that increasing the solubilities of OCP [7] and HA [80,81,82,83] enhances the osteoblastic differentiation of MSCs and osteoblast-like cells in vitro. In the OCP/collagen composites, OCP granules with a diameter of 300–500 μm progressed a hydrolysis reaction more compared to those with a diameter of 53−300 μm [18]. In the present study, the ALP activities were significantly higher in the OCP and HA < 53 μm groups than in the OCP and HA 300–500 μm groups, respectively (Figure 5). The Ca^2+^ induced by the hydrolysis of OCP and apatite formation on HA seemed to be inhibited in the <53 μm groups compared to the 300–500 μm groups (Figure 6, Table 1). However, the difference in the Pi concentration among the granular sizes was not observed clearly because of the decomposition of β-glycerophosphate and ascorbic acid 2-phosphate by the cellular metabolism [6,84].

The morphological observations indicated that the iPSC spheroid formation process in the <53 μm groups was similar to that of the MSC hybrid spheroids including the granules with a diameter of <53 μm [59]. The inclusion of CaP granules with a diameter of <53 μm in MSC spheroids has previously been confirmed through histological observation of the spheroids [59]. The previous studies confirmed by histological analysis that small particles with diameters of <53 μm, 3−5 μm, and 60 ± 50 μm are included homogeneously in the spheroids [59,72,73]. In contrast, the spheroids including no granules were also observed in the 300–500 μm groups (Figure 4), suggesting that the granules with larger diameters could be unsuitable to incorporate into the iPSC spheroids. The 3D cell–cell and cell–materials interactions are an important factor of spheroid formation [6,85,86,87,88], and the cell adhesion onto CaP materials can be enhanced on the larger specific surface area [18,89,90]. The specific surface area of OCP also increases with decreasing granule size if the granules contain the primary particle with the same morphology [18]. However, the part of larger granules exposed in the culture media could progress the reactions compared to the smaller granules inside of the iPSC spheroids. We also examined the solubility of OCP with different granular sizes in the culture media without cells (Table 2). The dissolution of Ca^2+^ and Pi ions tended to increase due to the decrease in the granules at an early stage of incubation. The higher DS values with respect to HA indicated that the medium incubated with the OCP granules with a diameter of <53 μm has a higher potential to progress HA transformation. In the previous study, imaging analysis of pH changes using the gel sheet displayed that the Pi concentration locally increased around the surface of OCP granules [18]. Thus, the smaller granules could be able to simulate the extracellular Pi transporter [18,76,91,92] of cells homogeneously inside the spheroids, resulting in the OCP granules with a diameter of <53 μm significantly promoting the osteoblastic differentiation of iPSC spheroids on the Oxy chip.

The 3D cultures of iPSCs have been applied for the screening of medicines and elucidation of biological functions [31,93,94,95,96,97,98,99,100,101,102,103,104,105]. We previously demonstrated that the ALP activity and osteopontin gene expression of mouse bone marrow-delivered MSC spheroid-including granular CaP materials (<53 μm) cultured on the Oxy chip positively correlated with the new bone volume in a critical-sized mouse calvarial defect treated with CaP granules (500−1000 µm) [6]. The new bone formation capacities of granular CaP materials in mouse calvarial defects increase in the following order: HA< β-TCP < OCP [6]. In the present study, mouse gingival-derived iPSC spheroids, including OCP < 53 μm material, showed the highest ALP activity, and ALP in HA < 53 μm material tended to be higher than in β-TCP < 53 μm material (Figure 5B). In contrast, there was no significant difference in the ALP activity of iPSC spheroids among the CaP materials with a diameter of 300–500 µm. The tendency in ALP activities of iPSC spheroids in the CaP < 53 μm groups could be in accord with the in vivo bone formation reported partially in the previous study. There are some limitations in the present study: (1) because ALP is an undifferentiation marker of iPSCs [61,62,106,107], an analysis of the gene expression of another osteoblastic differentiation maker is needed; (2) an analysis of which stage of osteogenic differentiation, including the calcification of iPSCs [46,69], is enhanced by OCP should be performed; (3) an analysis of which inorganic ions have an effect on the MSC differentiation is required; (4) an analysis of whether iPSC sources affect MSC differentiation by OCP is required; (5) analysis about the possibility of the use of the CaP materials and iPSC-derived MSC spheroids can be applied to bone defects in an animal study is needed. The overall results of the present study indicate that local inorganic ion concentration changes [18] around the smaller OCP granules could stimulate the osteoblastic differentiation of iPSCs inside the spheroids cultured using the Oxy chip. This suggests that supplying oxygen and providing the homogeneous incorporation of granules inside the cell aggregation could be important factors in simulating the in vivo osteogenesis induced by CaP materials utilizing an iPSC spheroid culture in vitro.

## 5. Conclusions

The present results suggest that OCP enhances the osteoblastic differentiation of a hybrid spheroid consisting of mouse gingival-derived iPS cells and OCP assembled three-dimensionally. Although the clinical application of OCP has just begun with OCP/collagen in dentistry [1] and OCP/gelatin [108] in orthopedics, it is expected that bone regeneration therapy could be established by combining OCP with cells, focusing on the bioactivity of OCP revealed in the present study. It is also required to analyze the interaction between OCP and serum proteins/growth factors [5,47], elucidating the osteogenic performance of OCP bone substitute materials.

## Figures and Tables

**Table 1:** Solution composition and degree of supersaturation (DS) of culture medium before and after the cultivation of iPSCs and CaP materials in Oxy chip.

Supernatants	Periods (Days)	DS at 37 °C		
		HA	OCP	DCPD
Osteogenic medium	0	5.30 × 10^14^	1.11 × 10^5^	1.04 × 10^0^
				
iPSCs only (Oxy chip)	2	6.97 × 10^12^	2.02 × 10^4^	1.41 × 10^0^
OCP < 53 μm	2	5.97 × 10^12^	2.36 × 10^4^	1.65 × 10^0^
OCP 300–500 μm	2	3.31 × 10^13^	6.66 × 10^4^	1.86 × 10^0^
HA < 53 μm	2	1.74 × 10^13^	4.94 × 10^4^	1.89 × 10^0^
HA 300–500 μm	2	1.50 × 10^13^	3.85 × 10^4^	1.69 × 10^0^
β-TCP < 53 μm	2	8.37 × 10^13^	1.63 × 10^5^	2.48 × 10^0^
β-TCP300–500 μm	2	3.05 × 10^13^	6.18 × 10^4^	1.82 × 10^0^
				
iPSC (Oxy chip)	4	1.80 × 10^13^	6.02 × 10^4^	2.13 × 10^0^
OCP < 53 μm	4	1.27 × 10^13^	4.88 × 10^4^	2.08 × 10^0^
OCP 300–500 μm	4	8.22 × 10^12^	3.30 × 10^4^	1.85 × 10^0^
HA < 53 μm	4	3.70 × 10^13^	9.48 × 10^5^	2.27 × 10^0^
HA 300–500 μm	4	7.95 × 10^12^	2.60 × 10^4^	1.60 × 10^0^
β-TCP < 53 μm	4	3.09 × 10^14^	4.66 × 10^5^	3.24 × 10^0^
β-TCP 300–500 μm	4	3.76 × 10^13^	7.75 × 10^4^	1.97 × 10^0^
				
iPSCs only (Oxy chip)	6	7.78 × 10^13^	1.47 × 10^5^	2.37 × 10^0^
OCP < 53 μm	6	3.88 × 10^13^	9.88 × 10^4^	2.30 × 10^0^
OCP 300–500 μm	6	2.56 × 10^12^	9.48 × 10^3^	1.19 × 10^0^
HA < 53 μm	6	5.09 × 10^13^	9.33 × 10^4^	2.02 × 10^0^
HA 300–500 μm	6	1.43 × 10^11^	8.69 × 10^2^	6.33 × 10^−1^
β-TCP < 53 μm	6	2.03 × 10^14^	2.66 × 10^5^	2.56 × 10^0^
β-TCP 300–500 μm	6	3.55 × 10^13^	5.82 × 10^4^	1.66 × 10^0^
				
iPSC only (Oxy chip)	14	9.25 × 10^12^	2.77 × 10^4^	1.59 × 10^0^
OCP < 53 μm	14	9.68 × 10^12^	3.04 × 10^4^	1.66 × 10^0^
OCP 300–500 μm	14	1.50 × 10^12^	6.11 × 10^3^	1.06 × 10^0^
HA < 53 μm	14	6.58 × 10^12^	1.99 × 10^4^	1.43 × 10^0^
HA 300–500 μm	14	2.02 × 10^12^	7.67 × 10^3^	1.12 × 10^0^
β-TCP < 53 μm	14	8.44 × 10^12^	2.56 × 10^4^	1.55 × 10^0^
β-TCP 300–500 μm	14	2.58 × 10^13^	5.04 × 10^4^	1.68 × 10^0^

**Table 2:** Ion compositions and DS with respect to CaP phases in the culture media after incubations of OCP granules without iPSCs.

Supernatants	Periods (Days)	Ca (mM)	Pi (mM)	pH	DS at 37 °C		
					HA	OCP	DCPD
Original medium	0	2.46	1.23	7.92	5.30 × 10^14^	1.11 × 10^5^	1.04 × 10^0^
							
OCP < 53 μm	2	1.56	0.81	7.74	3.24 × 10^12^	2.40 × 10^3^	4.41 × 10^−1^
	14	1.61	0.95	7.36	1.27 × 10^11^	5.24 × 10^2^	4.71 × 10^−1^
							
OCP 300–500 μm	2	1.40	0.77	7.83	4.02 × 10^12^	2.19 × 10^3^	3.86 × 10^−1^
	14	1.33	0.93	7.37	5.26 × 10^10^	2.51 × 10^2^	3.87 × 10^−1^

## Data Availability

Data supporting the findings of this study are within this manuscript.
